# Self-Efficacy and Perceived Stress in Women Experiencing Preterm Birth

**DOI:** 10.3390/jcm13164945

**Published:** 2024-08-22

**Authors:** Agata Białas, Anna Nowak, Karolina Kamecka, Paweł Rasmus, Dariusz Timler, Michał Marczak, Remigiusz Kozłowski, Anna Lipert

**Affiliations:** 1 Department of Preventive Medicine, Medical University of Lodz, 92-213 Lodz, Poland; agawhite@znajomi.pl (A.B.); anna.nowak10@stud.umed.lodz.pl (A.N.); 2 Department of Management and Logistics in Healthcare, Medical University of Lodz, 90-131 Lodz, Poland; kkamecka@gmail.com (K.K.); michal.marczak@umed.lodz.pl (M.M.); remigiusz.kozlowski@umed.lodz.pl (R.K.); 3 Department of Medical Psychology, Medical University of Lodz, 90-131 Lodz, Poland; pawel.rasmus@umed.lodz.pl; 4 Department of Emergency Medicine and Disaster Medicine, Medical University of Lodz, 90-419 Lodz, Poland; dariusz.timler@umed.lodz.pl

**Keywords:** mother, premature infant, mother–infant relation, emotions, coping inventory, stressful situations, anxiety

## Abstract

**Background:** Being an unexpected, undesired and life-threatening situation, preterm birth (PTB) is a stress-, anxiety- and depression-generating factor for women delivering prematurely. The aim of this study was to assess the relationship between self-efficacy, coping strategies and perceived stress in mothers who experienced preterm birth and full-term birth, to determine the needs for personalized emotional support. **Methods:** The study was conducted among 251 women divided into the preterm birth group (PBG) and the full-term birth group (FBG). Data were collected using the following: (1) The State-Trait Anxiety Inventory (STAI) Questionnaire, (2) Generalized Self-Efficacy Scale (GSES) and (3) Coping Inventory for Stressful Situations Questionnaire (CISS), which were distributed online from January 2021 to June 2021. **Results:** Lower STAI scores were recorded in the preterm birth group (PBG) with high self-efficacy (HSE) when compared to the full-term birth group with HSE. CISS test scores were higher in PBG women with low self-efficacy (LSE) in comparison to women with LSE in FBG (*p* < 0.001). A positive and strong relationship (0.83; *p* < 0.05) was found between avoidance-oriented style and strategy of avoidance by engaging in surrogate activities and a positive moderate relationship (0.58; *p* < 0.05) with the style of looking for social contacts in PBG with LSE. **Conclusions:** The task-oriented coping style seems to be the most beneficial strategy for mothers, regardless of their preterm or term delivery, as focusing on specific activities increases the sense of self-efficacy and the anxiety level can decrease. Awareness of different styles of coping with stress and a sense of self-efficacy are necessary to plan personalized interventions for premature infants’ mothers.

## 1. Introduction

In 2020, 13.4 million babies were born preterm [1], while complications of preterm birth (PTB) were reported to be the leading cause of death among children under 5 years old [2]. As an unexpected, undesired and life-threatening situation, PTB is a stress-, anxiety- and de-pression-generating factor for women delivering prematurely [3,4]. Moreover, the maternal emotional state and hospital environment contribute to the disruption of the normal mother–infant physical contact and exert an influence on maternal care and mother–infant bonding [5].

The constant and worldwide promotion of the multi-aspect definition of health according to the Constitution of the World Health Organization is a crucial aspect in healthcare. Mental health is an integral part of health and well-being and, like all other aspects of health, can be affected by a range of factors [6]. Yet, it remains a taboo topic, which causes people to be ashamed and unwilling to talk about the issue. At the same time, help in mental health deficits is most needed, especially in providing care for a newborn in a highly demanding situation such as a premature delivery.

Self-efficacy has been one of the important psychological aspects studied in women during pregnancy and postpartum [7,8,9,10,11]. The concept of self-efficacy comes from Albert Bandura’s social learning theory. Self-efficacy is a personal belief in one’s own ability to succeed in specific situations, to achieve set goals or to accomplish a task [12,13,14]. Personal expectations regarding self-efficacy may determine whether or not to undertake certain behaviors. The self-efficacy concept is of foremost importance as an element of mental health, as this personal resource affects our way of coping with stress. The assessment of self-efficacy level has an impact on the scope, form and personalization of the psychological support offered to people in need and the individual self-efficacy level determines potentially recommended psychological interventions. The relationship between social support, self-efficacy and characteristics of research groups has already been studied [15,16].

Self-efficacy is strongly related to perceived stress and, therefore, anxiety. In difficult and stressful situations, stress causes significantly lower self-efficacy, which leads to the fear of not being able to achieve the intended goal. Perceived stress reflects an individual’s subjective experience of the stress they feel at a particular moment or over a period of time. This perception can vary based on factors such as coping mechanisms and available support networks [17].

The successful development of preterm infants has been reported to depend, among others, on the mother’s mental state [15]. Premature newborns’ mothers do not have favorable conditions to achieve their goal, i.e., a full-term delivery, nor do they have any impact on treatment and prognosis, which is why psychological support for such mothers is so necessary. The support for mothers of preterm infants should be personalized, i.e., coping failure in the mental aspect which can be observed by medical staff should result in specific and relevant assistance. The assessment of anxiety and stress coping styles in mothers of premature babies may be a key tool in the effective selection of information and further support methods.

Task-focused coping addresses the sources of stress in practical ways, alleviating the stress-causing event or circumstance, and relieving stress. It aims to reduce or even eliminate the causes of stress through methods such as problem-solving, time management, and receiving instrumental social support [18]. In contrast, emotion-oriented coping focuses on managing emotional responses to stressors. This can involve reframing the issue to diminish its negative emotional impact and alleviate stress. Finally, avoidance-oriented coping encompasses activities and cognitive strategies like avoiding or denying the situation, as well as indirect approaches such as distancing oneself or engaging in unrelated activities to lessen stress levels [19].

Recommendations for the care provided to preterm or low-birth-weight infants include guidance on providing emotional, financial and workplace support to the families of premature newborns [20].

Interpretive description studies underscore the traumatic nature and resultant psychological distress related to a traumatic event such as PTB. This traumatic experience has a number of in-depth consequences on parents’ mental condition [21]. While the importance of mental health is widely acknowledged, this study delves deeper into how specific coping strategies employed by mothers during PTB relate to their self-efficacy. Based on current knowledge, there is still not enough information and research focusing on the coping strategies, anxiety levels and mental health in general of women experiencing preterm birth. The available literature has some limitations, such as a small sample size or not comparing data such as anxiety levels between different groups [22,23].

The issue of psychological maternal care at PTB as well as the focus on mother–preterm and mother–environment relationships is a highly urgent task in the process of perinatal care. By identifying coping strategies and understanding their implications, we can address the unique needs of mothers facing PTB. Fowler C. et al. noticed that the mothers of extremely preterm babies were a high-risk group for the development of post-traumatic stress symptoms or mental health disorders in general [24]. Although the development of supportive initiatives for experienced preterm children’s mothers has been identified as necessary for the prevention of mothers’ mental health disorders [25], first, a greater understanding is needed of the point of view and emotions of preterm infants’ mothers and those data are still limited, inconclusive or outdated [26,27,28]. We hypothesized that the level of anxiety among preterm infants’ mothers can be influenced by the level of self-efficacy. In addition, presenting the specific style of coping with stress may also be important. Therefore, the aim of this study was to explore the relationship between self-efficacy, coping strategies and perceived stress in mothers who experienced preterm birth and full-term birth to determine the needs for personalized emotional support.

## 2. Materials and Methods

### 2.1. Study Design and Procedure

This was an exploratory study conducted as a diagnostic survey. Data were collected among the female patients in several clinical hospital around the country which had neonatal care or neonatal intensive care units. Two hundred and fifty-one women were included in the study, of which one hundred and twelve of whom delivered prematurely and one hundred and thirty-nine mothers who delivered infants at term. Division was made for PBG and FBG, of which both had to be characterized by relatively similar socio-demographic characteristics. The sole difference was that PBG included mothers who gave birth to children prematurely (when infants were born before 37 weeks of gestation). The newborns were hospitalized in a variety of centers throughout Poland.

In our study, we used specific inclusion criteria.

The following inclusion criteria were applied to the two groups of women:

- PBG: (1) infants born before 37 weeks of gestation, (2) hospitalization duration not shorter than one month and no longer than 6 months, (3) timespan after discharge from hospital not longer than 2 months, (4) natural birth, (5) no genetic defects in preterm infants.
- FBG: (1) infants born after 37 weeks of gestation, (2) duration of hospital stay not longer than 3 days, (3) timespan after discharge from hospital not longer than 2 months, (4) natural birth, (5) parents of newborns with no genetic defects.

### 2.2. Methods

The following research tools were used to collect data: (1) The State-Trait Anxiety Inventory (STAI) Questionnaire—a 20-question tool used in research as an indicator of stress [29] to diagnose and distinguish the situation-related anxiety and anxiety as a feature from depressive syndromes [30,31]; (2) Generalized Self-Efficacy Scale (GSES)—a 10-statement scale diagnosing a general sense of perceived self-efficacy in handling difficult situations and life hurdles [32,33]; (3) Coping Inventory for Stressful Situations Questionnaire (CISS)—a 45-statement tool used to define stress coping strategies (Task-Oriented, Emotion-Oriented, Avoidance) [34,35,36,37] and describe human behavior in stressful situations; (4) original survey questionnaire developed to obtain the characteristics of the study population of preterm infants’ mothers and the tool collected socio-demographic data.

STAI is used to diagnose both state anxiety (situation-related anxiety) and trait anxiety as a relatively permanent personality trait (anxiety as a feature) [30]. The questionnaire is divided in two parts, each including 20 questions pertaining to state anxiety (Questionnaire X1) and trait anxiety (Questionnaire X2). The participants rate themselves on each question using a 4-point frequency scale. Questionnaire X1 includes 10 direct and 10 indirect questions while Questionnaire X2 consists of 13 direct questions and 7 indirect ones. If both parts are used in research, questionnaire X1 is recommended to be used as the first one. The total scores were calculated by adding up the scores of each item worded directly and reverse scoring is used for indirect questions.

GSES is used to assess a general sense of perceived self-efficacy in handling difficult situations and life hurdles [32]. The scale consists of 10 statements which are part of one factor and the results are calculated according to the key that should be interpreted by the means of the scores. The internal reliability scores of the Polish version of GSES are reported to be good with a Cronbach alpha coefficient = 0.85 [32].

CISS consists of 48 statements describing human behavior in stressful situations [35]. In CISS, the score is calculated in three 16-item scales: T, E and A. The total of the points in each scale is the raw result. The sores range from 16 to 80 points. The Avoidance score can also be calculated in two subscales: A–D (8 items) and A–SD (5 items). Respondents self-assess their behavior using a 5-point Likert scale describing the frequency of a certain coping method used in a stressful situation. The available Polish version of the inventory was developed by P. Szczepaniak, J. Strelu and K. Wrześniewski in the 1990s. The questionnaires were distributed online due to COVID-19 pandemic. The data were collected in the period from 15 January 2021 to 15 June 2021. The survey questionnaires were available and distributed online. The online form of data collection was the only method of reaching the study group allowed by healthcare facilities with COVID-19 being the data collection period. Completing the questionnaires was anonymous and equivalent to agreeing to participate in the study. The survey questionnaire included information that participation in the study was voluntary and that the person could resign from it at any time. The study obtained approval from the local University Human Ethics Committee (RNN/170/21/KE).

### 2.3. Statistical Analysis

Statistical analyses were performed using Statistical version 13.1 software (StatSoft, Tulsa, OK, USA). The data were not normally distributed (Shapiro–Wilk test for normal distribution analysis), so the Mann–Whitney test was used to analyze the differences between the groups. The data were presented as the median and confidence intervals of the mean or number of people and the percentages were applied. The Spearman correlation was performed to analyze the relationship between the results of psychological tests and the self-efficacy level. Significant differences were accepted for all analyses at the level of *p* < 0.05.

## 3. Results

### 3.1. Characterisctics of the Study Group

The study involved 251 women who were divided into the preterm birth group (PBG) and the full-term birth group (FBG). The two subgroups were distinguished in both groups according to the self-efficacy level. Most of the analyzed women were married, both in the PBG and FBG groups. Almost half of all the studied women (both groups) with a high self-efficacy (HSE) level came from a city with over 50,000 citizens, while the ones with a low self-efficacy level (LSE) came from both cities and villages. Most of the women had a high level of education (Table 1).

### 3.2. Psychological Analysis

In general, more significant differences in psychological tests results were found between the PBG and FBG groups in women with lower self-efficiency scores (Table 2). Higher scores for the STAI test were obtained by PBG women with LSE in comparison to FBG women with LSE. However, lower STAI scores were recorded in PBG women with HSE compared with FBG women with HSE (Table 2 and Table 3). The CISS-E test scores were higher in PBG women with LSE in comparison to FBG women with LSE, with *p* < 0.001 (Table 2), which indicates higher anxiety levels in the former. Moreover, the scores obtained in CISS in avoidance strategy by distraction (A–D) were slightly more elevated than the results recorded in FBG women with LSE. This may mean that PBG women with HSE represented a style focused on avoidance by engaging in surrogate activities more than FBG women with LSE (Table 2). The same finding was observed in PBG women with HSE in comparison to FBG women with HSE (Table 3). However, PBG women with HSE used less task-oriented strategy in comparison to C women with HSE, and the difference was statistically significant (*p* < 0.05). Moreover, both PBG and FBG women with HSE obtained lower STAI test results, which suggests they experienced less anxiety in comparison to PBG and FBG women with LSE (Table 2 and Table 3).

### 3.3. Spearman Correlation Results

A positive and strong relationship (r = 0.83; *p* < 0.05) was found between avoidance-oriented style and strategy of avoidance by engaging in surrogate activities, and a positive moderate relationship (r = 0.58; *p* < 0.05) with the style of looking for social contacts in PBG women with LSE (Table 4). The emotion-oriented coping style was negatively but insignificantly related to (r = −0.35; *p* < 0.05) the task-oriented style, and positively linked to (r = 0.40; *p* < 0.05) the strategy of avoidance by engaging in surrogate activities (Table 4).

Similar relationships were observed in PBG women with HSE; however, their style focused on avoidance was positively correlated (r = 0.29; *p* < 0.05) with the style concentrated on emotions (Table 5). Also, a positive relationship (r = 0.43; *p* < 0.05) was observed between the emotion-oriented strategy and avoidance by distraction strategy in coping with stress. (Table 5).

## 4. Discussion

Our study revealed several important insights into the coping strategies of mothers with preterm infants. However, due to the state of the prevailing pandemic, we were forced to collect our data online. We assume that a negligible number of mothers may have been in the hospital at the time and participated in the study online. Not being able to anticipate a situation such as a pandemic, in planning our study, we focused on what we considered most relevant in the time before the pandemic. The strictly defined length of time a child was in the hospital seemed to us to be an important factor. Therefore, in the study’s inclusion criteria, in order to standardize the study group, we asked that the child’s time in the hospital should be no less than one month and no more than six months, and the possible time since the child left the hospital should not exceed two months. The very aspect of the possibility (or lack thereof) of mothers staying in the pandemic period with a premature child during their hospitalization is undoubtedly an important and necessary topic, so we are considering the possibility of looking at this aspect in the future.

Focusing on the anxiety levels, higher scores were recorded in our study’s preterm infants’ mothers with LSE than in women with LSE who delivered at term. Early postnatal maternal separation and specific environment of neonatal intensive care units (NICU) were demonstrated [38,39] to induce stress and adversely affect the emotional and mental state of parents, mothers in particular. Consistently, our findings confirmed that unfavorable environment of the hospital ward, the newborn’s condition, invasive procedures, the infant’s pain and stress, and consequently, parental fear for their babies’ life and health had a negative impact on the anxiety level in preterm infants’ mothers and infants at neonatal intensive care units.

With respect to coping styles, our investigation revealed that the low self-efficacy level of mothers in both preterm and full-term birth groups was, to a large extent, related to coping strategies by distraction and social diversion. However, a considerable difference was detected between the two LSE groups in the emotion-oriented strategy of coping with stressful situations, which is all the more noteworthy if we intend to indicate solutions for personalized interventions.

The coping style focused on experiencing emotions seems to be inextricably linked with stressful situations which accompany preterm delivery. Uncertain infant’s health status and prognosis tend to aggravate maternal anxiety and invoke a reduced sense of self-efficacy. The outcomes of our study consistently confirmed that preterm infants’ mothers with LSE who experienced the preterm delivery hardship mainly resolved to emotion-oriented coping strategies, and they engaged in alternative tasks that resulted from helplessness and a higher anxiety level. Such maternal emotions and behaviors were also reported in other available studies [40]. Therefore, relevant help should be offered and implemented as a matter of urgency.

Participants who used emotion-oriented coping strategies showed a negative minor relationship with the task-oriented style and a positive correlation with avoidance by the distraction strategy. Available literature describes the emotion-oriented coping style as more frequently used by prematurely delivering women [38]. Adequately to these findings, the analysis of our study’s data on low efficacy in preterm infants’ mothers revealed their high level of the emotion-oriented coping style. Similar findings were reported by Mariański et al., where the increase in the emotion-oriented style of coping with stress was shown to be positively correlated with the sense of hopelessness in mothers of preterm infants. The concept of hopelessness is close to the scientific term used in our analysis, i.e., self-efficacy. Mariański et al. also emphasized that the sense of hopelessness correlated positively with the emotion-oriented style in both investigated groups.

In our study, the preterm infants’ mothers with HSE displayed a less task-oriented coping style than full-term infants’ mothers with HSE, and the difference was statistically significant. Furthermore, our study shows that the applied test results between preterm and full-term infants’ mothers were more substantial in women with LSE. In this study, the avoidance-oriented coping style was found to correlate most strongly with distraction and social diversion strategies in mothers with low self-efficacy scores. Further investigation is worthy to analyze whether seeking social contacts is more connected with the search for emotional support from close relatives or medical personnel that could provide answers related to the infant’s health status and indicate the best form of support. The available literature reported that the avoidance-oriented coping style involved avoidance of thinking of the cause of stress and, as a consequence, distraction from the adversity, its potential consequences and one’s own emotions by focusing on other activities [21,22,23,24,25,26,27,28,29,30,31,32,33,34,35,36,37].

The most considerable difference was also observed between the preterm birth and full-term birth groups with high self-efficacy scores in the emotion-oriented style of coping with stress. In this high self-efficacy group, social diversion and distraction strategies were found to be closely interrelated and corelated with the emotion-oriented style. Therefore, avoidance of thinking of the adversity, engagement in alternative tasks and seeking social contacts were strictly connected with experiencing emotions, regardless of the self-efficacy scores.

The task-oriented strategy of coping with stress is characteristic of people who primarily strive to identify the source of the problem and then seek solutions. If the issue proves unsolvable, they try to adjust to the new situation. The strategy is advantageous as it allows for a prompt and effective response to the hardship to begin to cope with the cause of stress. However, the situations that are beyond our control and the problems to which the solutions are yet to be found belong to exceptions. Preterm infants’ mothers are undoubtedly in such situations and although half of our participants engaged in the task-oriented strategy, the other half succumbed to the sense of helplessness, strong emotions and surrogate activities [34,35].

It is hardly surprising that anxiety accompanies mothers when their newborns’ life is at risk, and our study demonstrated various coping strategies applied under the influence of anxiety. The more severe the stress, the more prevailing the strategy which is based on the emotions and avoidance of confrontation with the adverse event, which in practice leads to distraction and social contact seeking as a result. Such an interrelation was detected in mothers with both low and high self-efficacy scores, irrespective of the delivery: preterm or at term. What is also connected is the sense of self-efficacy in preterm infants’ mothers and social support. Thus, it should be taken into account by the nurses who provide the care for preterm infants so that they could apply strategies [41] to fulfill such mothers’ needs in a potentially most beneficial way. Both preterm birth and full-term groups included a comparable number of mothers who applied the task-oriented coping style, regardless of their self-efficacy scores.

A limited number of recent studies discussed the sense of self-efficacy and perceived stress by mothers of preterm infants. The available studies emphasized the need to involve healthcare professionals to support preterm infants’ mothers emotional state by practice and early discharge programs [42]. Focusing on the experience of preterm infants’ mothers after hospital discharge seems to play a crucial role in the development of educational strategies and counselling interventions in accordance with the mothers’ needs. Addressing maternal emotional well-being and enhancing the development of mother–preterm infant relationship should be an early intervention [43].

Studies show the importance of neonatal intensive care unit professionals in the process of optimizing the care for mothers immediately after the preterm birth and during the infant’s hospitalization, taking into consideration the psychological needs of mothers of preterm infants [22] and some implications for clinical practice to reduce the negative emotional toll on mothers of NICU babies [44].

The strength of our study is that it covers the gap in the national research on preterm infants’ mothers. It confirms that the styles of coping with stress used by preterm infants’ mothers and their sense of self-efficacy have an impact on the process of caring for a premature infant.

This study contributes to Polish scientific literature by providing evidence-based research results on self-efficacy and the perceived stress of women experiencing preterm birth. Our study was based on validated research tools that were also used in other studies [29,33,34,35,36,37]. The strength of the study is that the data were collected during the COVID-19 pandemic, because mothers of premature babies could not be visited personally due to government restrictions. This was the reason for online data collection, which probably resulted in a higher responsiveness rate than could have been the result of direct contact and attempts to meet each mother. Due to complete anonymity, without the need for direct contact with the researcher, mothers were encouraged to be more active in filling out the research questionnaires. However, a self-report online study also has some limitations, such as participants misunderstanding the questions or answering the questions in a way that they think makes them look good, especially if mental health—very sensitive issue—is assessed. Therefore, further studies based on additional direct tools and methods to obtain the data would be recommended. Another limitation is the small size of the study group; however, it is the consequence of the specialized nature of the research issue. Due to the fact that the similar previous research is limited, the sample size was also difficult to calculated in advance, so the present study is more exploratory rather than experimental. Therefore, the results should be presented with less certainty and more as a basis for future studies with larger sample sizes and better control over the information. Also, the participation of healthcare specialists to analyze their difficulties and relations with premature infants’ mothers would be valuable.

## 5. Conclusions

The task-oriented coping style seems to be the most beneficial strategy for mothers, regardless of their preterm or term delivery, as focusing on specific activities increases the sense of self-efficacy and the anxiety level can decrease. However, shaping a high level of self-efficiency, regardless the stress coping style, may also result in a decrease in anxiety levels.

Psychological support for preterm infants’ mothers based on their individual mental resources may enhance their sense of self-efficacy, help toward the decrease in stress and anxiety, and positively affect the care of preterm infants. Awareness of different styles of coping with stress and a sense of self-efficacy are necessary to plan personalized interventions for premature infants’ mothers.

## Figures and Tables

**Table 1:** Characteristics of the study participants.

Variable	PBG	FBG	*p*-Value	PBD	FBG	*p*-Value
	LSE (N = 49)	LSE (N = 40)		HSE (N = 63)	HSE (N = 98)	
Age Median (95%CI)	35.0 (33.1; 36.3)	35.0 (33.6; 37.9)	0.623	36.0 (34.9; 38.5)	38.5 (37.9; 40.9) *	0.041
Marital status N (%)						
Miss/bachelor	6 (12.2)	3 (7.5)	0.504	12 (19.0)	7 (7.1)	0.278
Married	40 (81.6)	33 (82.5)	0.496	45 (71.4)	81 (81.8) **	0.001
Divorced	3 (6.1)	4 (10.0)	0.797	6 (9.5)	9 (9.1)	0.489
Separated	0	0	1.000	0	2 (2.0)	0.259
Place of living N (%)						
City over 50,000 citizens	19 (38.8)	14 (35.0)	0.560	36 (57.1)	53 (53.5) *	0.035
City below 50,000 citizens	12 (24.5)	13 (32.5)	0.887	13 (20.6)	25 (25.2)	0.065
Village	17 (34.7)	12 (30.0)	0.508	14 (22.2)	19 (19.2)	0.410
No answer	1 (2.0)	1 (2.5)	1.000	0	2 (2.0)	0.259
Educational status N (%)						
Vocational education	0	0	1.000	2 (3.2)	1 (1.0)	0.597
Secondary education	7 (14.2)	4 (10.0)	0.542	11 (17.4)	11 (11.1)	1.000
Higher	42 (85.7)	36 (90.0)	0.705	50 (79.4)	87 (87.9) **	0.001

Abbreviations: PBG—Preterm birth group; FBG—full-term birth group; LSE—low self-efficacy; HSE—high self-efficacy; N—number of respondents; %—percentage; CI—confidence interval; statistical significance: * *p* < 0.05; ** *p* < 0.001.

**Table 2:** The results of the psychological tests in the study participants with lower values of self-effectiveness.

	PB G (N = 112)	FB G (N = 139)	
	Median (95% CI)		*p*-Value
CISS SSZ	58.0 (8.1; 12.2)	60.0 (8.4; 13.2)	0.501
CISS SSE	4.0 (1.2; 1.7)	46.0 (10.5; 16.5) **	0.0001
CISS SSU	40.0 (8.1; 12.1)	37.0 (7.3; 11.5)	0.632
CISS SSU ACZ	17.0 (5.1; 7.6)	16.5 (4.3; 6.8)	0.614
CISS SSU PKT	15.0 (3.1; 5.2)	15.0 (3.9; 6.1)	0.977
STAI	103.0 (20.4; 30.5)	100.0 (24.7; 38.8)	0.426

Statistical significance: ** *p* < 0.001.

**Table 3:** The results of the psychological tests in the study participants with higher values of self-effectiveness.

	PBG (N = 112)	F B G (N = 139)	
	Mediana (95%CI)		*p*-Value
CISS SSZ	56.0 (9.6; 13.7)	59.0 (8.2; 10.9) *	0.037
CISS SSE	4.0 (6.5; 9.3)	41.0 (11.1; 14.7) **	0.0001
CISS SSU	36.0 (6.9; 9.9)	37.0 (7.4; 9.8)	0.248
CISS SSU ACZ	15.0 (4.4; 6.3)	15.0 (4.7; 6.2)	0.846
CISS SSU PKT	14.0 (3.5; 5.0)	15.0 (3.9; 5.2)	0.105
STAI	81.0 (16.2; 23.1)	83.0 (19.0; 25.2)	0.789

Statistical significance: * *p* < 0.05; ** *p* < 0.001.

**Table 4:** The results of the Spearman correlations of the psychological tests in the study participants with lower values of self-effectiveness.

	CISS SSZ	CISS SSE	CISS SSU	CISS SSU ACZ	CISS SSU PKT	STAI
CISS SSZ	-	**−0.35**	0.01	−0.06	0.27	−0.05
CISS SSE	**−0.35**	-	0.24	**0.40**	−0.15	0.19
CISS SSU	0.01	0.24	-	**0.83**	**0.58**	0.06
CISS SSU ACZ	−0.06	**0.40**	**0.83**	-	0.17	0.05
CISS SSU PKT	0.27	−0.15	**0.58**	0.17	-	−0.05
STAI	−0.05	0.19	0.06	0.05	−0.05	-

The statistically significant correlations are bolded.

**Table 5:** The results of the Spearman correlations of the psychological tests in the study participants with higher values of self-effectiveness.

	CISS SSZ	CISS SSE	CISS SSU	CISS SSU ACZ	CISS SSU PKT	STAI
CISS SSZ	-	**−0.35**	0.20	−0.01	**0.43**	0.18
CISS SSE	**−0.35**	-	**0.29**	**0.44**	−0.12	0.18
CISS SSU	0.20	**0.30**	-	**0.81**	**0.72**	0.24
CISS SSU ACZ	−0.01	**0.44**	**0.81**	-	**0.26**	0.14
CISS SSU PKT	**0.43**	−0.12	**0.72**	**0.26**	-	0.16
STAI	0.18	0.18	0.24	0.14	0.16	-

The statistically significant correlations are bolded.

## Data Availability

The data presented in this study are available on request from the corresponding author.
